# Serum Proteomic Changes in Dogs with Different Stages of Chronic Heart Failure

**DOI:** 10.3390/ani12040490

**Published:** 2022-02-16

**Authors:** Ahmet Saril, Meric Kocaturk, Kazumi Shimada, Akiko Uemura, Emel Akgün, Pinar Levent, Ahmet Tarik Baykal, Alberto Muñoz Prieto, Carlos Fernando Agudelo, Ryou Tanaka, Jose Joaquin Ceron, Jorgen Koch, Zeki Yilmaz

**Affiliations:** 1 Department of Internal Medicine, Faculty of Veterinary Medicine, Bursa Uludag University, Bursa 16059, Turkey; ahmettsarill@gmail.com (A.S.); merick@uludag.edu.tr (M.K.); vetpinarlevent@gmail.com (P.L.); zyilmaz@uludag.edu.tr (Z.Y.); 2 Department of Veterinary Surgery, Faculty of Veterinary Medicine, Tokyo University of Agriculture and Technology, Tokyo 183-8509, Japan; ryo@vet.ne.jp; 3 Laboratory of Veterinary Surgery, Department of Clinical Veterinary Medicine, Division of Veterinary Research, Obihiro University of Agriculture and Veterinary Medicine, Sapporo 080-8555, Japan; anco@vet.ne.jp; 4 Department of Medical Biochemistry, Acibadem University School of Medicine, Istanbul 34750, Turkey; emelc.akgun@gmail.com (E.A.); atbaykal@gmail.com (A.T.B.); 5 Clinic for Internal Diseases, Faculty of Veterinary Medicine, University of Zagreb, Heinzelova 55, 10000 Zagreb, Croatia; alberto.munoz@um.es; 6 Small Animal Clinic, Faculty of Veterinary Medicine, University of Veterinary and Pharmaceutical Sciences Brno, Palackého Tř. 1946/1, 612 42 Brno, Czech Republic; caragvet@seznam.cz; 7 Interdisciplinary Laboratory of Clinical Analysis, Interlab-UMU, Regional Campus of International Excellence, University of Murcia, Espinardo, 30100 Murcia, Spain; jjceron@um.es; 8 Department of Veterinary Clinical Sciences, Faculty of Health and Medical Sciences, University of Copenhagen, DK-1870 Frederiksberg, Denmark; koch@sund.ku.dk

**Keywords:** proteomic, mitral valve disease, heart disease, ACVIM, dogs

## Abstract

**Simple Summary:**

Canine MMVD is a progressive chronic disease with variable clinical signs, with some patients remaining completely asymptomatic while others develop CHF. Here, the aims of the pilot study were to evaluate serum proteins by proteomic analysis in dogs at different stages of chronic heart failure (CHF) due to degenerative mitral valve disease (MMVD), and how these proteins can change after a conventional treatment. Study revealed 157 different proteins; 11 were up- and 21 down-regulated at a statistically significant level in dogs with CHF compared to controls. Based on the bioinformatic analysis, protein–protein interactions between complement proteins, fibrinogen subtypes and others (albumin precursor, serpins, inter-alpha-trypsin inhibitor heavy chain, fetuin, clusterin, apolipoproteins, and alpha-glycoproteins) showed that pathophysiology of CHF seems to be more sophisticated than we had thought. These proteins are associated with several cellular, biologic, and metabolic processes such as immune-inflammatory responses, hemostasis, oxidative stress, and energy metabolism, which might be detrimental in the progression of canine CHF. Their molecular and biological functions as well as roles in the signaling pathways, such as inflammation, cadherin signaling, nicotinic acetylcholine receptor signaling and Wnt signaling make them possible biomarkers and therapeutic targets for the diagnosis and treatments in dogs with different stages of CHF.

**Abstract:**

MMVD, the most common cause of CHF in dogs, is a chronic disease with variable clinical signs, with some patients remaining asymptomatic while others develop CHF. Here, we aimed to evaluate serum proteins by proteomic analysis in dogs at different stages of CHF due to MMVD, and proteome behaviors after conventional treatment. A total of 32 dogs were divided equally into four groups—stage A (healthy/controls), stage B2 (asymptomatic), stage C and stage D (symptomatic)—according to the ACVIM consensus. Serum proteomes were evaluated using LC/MS-based label-free differential proteome analysis. The study revealed 157 different proteins; 11 were up- and 21 down-regulated in dogs with CHF compared to controls. In stage B2 dogs, angiotensinogen (AGT) was up-regulated, but immunoglobulin iota chain-like, lipopolysaccharide-binding protein, and carboxypeptidase (CPN) were down-regulated. In stage C dogs, complement C3 (C3) and inter-alpha-trypsin inhibitor heavy chain were up-regulated, but hemopexin, and actin-cytoplasmic-1 (ACT-1) were down-regulated. In stage D dogs, AGT was up-regulated, whereas tetranectin, paraoxonase-1, adiponectin and ACT-1 were down-regulated. A decrease in CPN, C3 and AGT and an increase in ACT-1 were observed after treatment of dogs in stage C. This pilot study identified that dogs at different stages of CHF show different serum protein composition which has potential to be biomarker for diagnose and treatment monitorization.

## 1. Introduction

Degenerative myxomatous mitral valve disease (MMVD) is the most common cause of chronic heart failure (CHF) in dogs. There is an increase in incidence with age, and most affected dogs are small breeds [1]. Progressive collagen degeneration and accumulation of acid mucopolysaccharide at the free edges of the mitral leaflets lead to thickening, deformation, and dysfunction of the valve and chorda tendinea [2,3]. The pathological changes give rise to mitral regurgitation leading to a gradually increasing chronic volume load on the left side of the heart, resulting in clinically detectable left atrial and/or left ventricular enlargement. In most of these dogs, the volume overload causes the development of signs of congestive heart failure such as pulmonary venous congestion and edema [4,5].

MMVD is a progressive chronic disease with variable clinical signs, with some patients remaining completely asymptomatic but others develop CHF. Thus, depending on the presenting symptoms, MMVD has been staged from the initial step (stage A: healthy dogs with a genetic predisposition) to the terminal phase of CHF (stage D: refractory CHF) by the American College of Veterinary Internal Medicine (ACVIM, Greenwood Village, CO, USA) consensus statement [6].

Serum concentrations of cardiac troponin-I (cTnI) and natriuretic peptides (NT-ProBNP) have been used as biomarkers of myocardial injury and ventricular wall stress, respectively, in dogs with heart disease [7,8]. However, these analytes may be influenced by non-cardiac problems, causing misconceptions in diagnosis and treatment choices [9]. Thus, there is an increasing interest in finding new and reliable biomarkers to evaluate the presence and severity of heart disease and to monitor treatment response in these cases.

Understanding the different pathophysiological mechanisms in different stages of CHF could contribute to disease prevention and development of individualized therapy in humans [10] and dogs with CHF [11]. In a previous study [11], we saw that there are changes in serum components in dogs at different stages of CHF due to MMVD, and that it would be of interest to evaluate whether there are more proteins that can change. Proteomics studies serve these purposes by identifying thousands of low-molecular-weight proteins in biological samples such as serum [12,13], myocardial tissue [14,15] and platelets [16], and determining protein–protein interactions [12,13,16].

Previous research using proteomic analysis of myocardial tissue has identified new pathophysiological mechanisms contributing to cardiovascular diseases such as ischemic cardiomyopathy [15], dilated cardiomyopathy (DCM) [14], diabetic cardiomyopathy [17,18], and atherosclerosis [19]. Recently, we showed changes in platelet proteins, such as clusterin, CXC-motif-chemokine, cathepsin, creatine-kinase-B and myotrophin, to be associated with cellular, biologic, metabolic, immune, and coagulation system processes involved in the development of CHF caused by MMVD in dogs [16]. Although there are limited studies on serum proteomics in dogs with MMVD [13], idiopathic DCM [20,21] and CHF [12], there are no data about possible changes in proteins depending on the severity of CHF associated with MMVD and the evolution of these proteins after standard treatment.

This study aimed to evaluate the composition of serum proteins by proteomics techniques at different stages of CHF due to MMVD in dogs, and how these proteins can change after conventional treatment.

## 2. Material and Methods

This prospective study was performed between July 2018 and January 2020 at the Veterinary Teaching Hospital, Bursa Uludag University (BUU), Bursa, Turkey, and approved by the Animal Experiments Local Ethics Committee of the BUU (ID: 2018-05/02).

### 2.1. Dogs

This study included dogs of different breeds (Labrador, Cavalier, Anatolian Shepherd, German Shepherd, Cocker Spaniel, Terrier, etc.), ages (3–11 years), and body weights (11–42 kg), as well as both sexes (17 males and 15 females). Groups of dogs used in this study were distinguished based on the results of physical, hematological and serum biochemistry examinations and cardiac imaging findings (Supplementary Files S1 and S2), which were compatible with the selection criteria for CHF from stage A to D described by ACVIM consensus guidelines for the diagnosis and treatment of MMVD in dogs [6].

### 2.2. Groups

Thirty-two dogs were divided equally into four groups in which some of these dogs with MMVD were the same as those in our recently published study on the changes in inflammatory and oxidative stress biomarkers in dogs with different stages of CHF [11]. Cases were grouped according to the ACVIM consensus statement [6]. Briefly, the dogs from predisposed breeds (3 Cavalier King Charles Spaniel, 2 Terrier, 2 Pekingese, and 1 Cocker Spaniel) that did not show the changes at the physical, clinical, and analytical examinations were categorized as stage A and used as healthy controls. The dogs who did not show a clinical complaint but had a systolic heart murmur over the mitral valve area and radiographic and echocardiographic evidence of cardiac remodeling due to MMVD were considered as stage B2 that refers asymptomatic phase of CHF. Dogs who displayed one or more clinical signs, such as coughing, hyporexia, tachypnea, and/or exercise intolerance in combination with a systolic murmur due to CHF caused by MMVD were categorized as symptomatic stages (stage C and D groups) of CHF, as reported in our [11] and other previous studies [12,13,22]. The group including stage B1 dogs (asymptomatic) was not included due to insufficient material during the specimen collection period.

If the dogs had a comorbid disease such as infectious, autoimmune, neoplastic, endocrine, and/or metabolic diseases, and received medication prior to admission to the clinic, they were not included to the study, because of the potential effects on serum proteomic profile. This was not valid for stage D dogs, because they must already be being treated with a standard protocol as described below, and are described as refractory CHF despite the treatment [6].

### 2.3. MMVD Definition

MMVD was diagnosed by trans-thoracic echocardiography including the following criteria: the presence of mitral valve prolapse (MVP) and/or thickening of the mitral valve anterior leaflet by 2-D echocardiography, and identification of mitral valve regurgitation by color Doppler examination as reported in our [11] and other previous studies [13,22].

### 2.4. Treatments

Dogs with stage A did not receive any medication, because they did not have problems with their cardiopulmonary system. Only pimobendan (0.25 mg/kg, twice daily, PO) was recommended to slow the progression of MMVD in stage B2 dogs. Conventional medical therapy was suggested for the dogs at stages C and D, which included a combination of pimobendan (0.25 mg/kg, q12 h, PO), furosemide (1–4 mg/kg, q12 h, PO), spironolactone (2–4 mg/kg, q24 h, PO), ACE inhibitor (enalapril: 0.5 mg/kg, q12 h, PO; or Ramipril: 0.125 mg/kg, q24 h, PO), and a supplementation product including Q10, taurine, carnitine, vitamin E and magnesium (CardioVet^®^ tablets, VetExpert, Lomianki, Poland). In dogs suffering from atrial fibrillation or sinus tachycardia, antiarrhythmic agents such as digoxin (0.005 mg/kg, q12 h, PO) and/or diltiazem (0.5–1.0 mg/kg, q8–12 h, PO) were used [6].

### 2.5. Measurements

Details of the physical (heart and respiratory rates etc.), hematological (complete blood cell counts) and serum biochemistry examinations (hepato-renal markers, electrolytes, cardiac troponin I—cTnI, and C reactive protein—CRP, etc.) of these dogs were described in our previous study [11].

#### 2.5.1. Cardiac Imaging

For the assessment of cardiac geometry and function, a transthoracic echocardiographic examination was performed as previously suggested [12,13,16]. Cardiac measurements were performed using conventional modalities (2-D, M-mode, and color Doppler) and imaging techniques (right parasternal short and long axis, left apical 4–5 chamber and subcostal views) with phased-array cardiac transducers (2.5–5, 5–7.5 or 7.5–10 MHz) selected according to body weight and the size of the dogs (Caris Plus Esaote, Genoa, Italy).

Although there are a lot of parameters measured during the standard echocardiographic examination, only some of them were selected, in line with the objectives of this study. Left atrial to aortic root ratio (LA/Ao) and the normalized left ventricular internal diameter at end diastole (LVIDDN) from right parasternal echocardiographic views were calculated [23]. Left atrial and left ventricular enlargement were considered when LA/Ao ratio and LVIDDN were greater than or equal to 1.6 and 1.7, respectively [6]. Fractional shortening (FS%) was calculated from left ventricular internal diameter at diastole (LVIDd) and systole (LVIDs) by 2-D M-mode measurements, at right parasternal long axis view [24]. Mitral early (E wave) and late diastolic flows (A wave) were obtained from the left parasternal window—apical 4–5 chamber view and measured by placing the sample volume on the top of the mitral valve leaflets using pulse wave Doppler [13,24].

Two weeks after the initiation of the medical therapy, all dogs were re-examined, but only data collected from dogs in stage C were used, to evaluate changes in the proteins after treatment. Stage D dogs were not preferred for this comparison, because the drugs used to relieve the clinical signs of CHF before being enrolled into the study could affect the protein composition in such dogs, as reported in our previous study [16].

#### 2.5.2. Proteomic Analysis

In this study, the current dog protein sequence information from NCBI and Uniprot databases was used as the protein database as performed in our previous study [21] relating to serum proteomes in dogs with DCM. Serum proteomic analysis was carried out by the LC-MS/MS method (Waters M-Class UPLC and Xevo G2-XS QTOF MS, Etten-Leur, The Netherlands). Albumin depletion from canine serum was evaluated following the salt–ethanol precipitation protocol from Bilic et al. [20]. Briefly, 100 µL aliquot of serum sample was mixed with 10 µL 1 M NaCI and incubated for an hour at 4 °C. Cold ethanol was added to a final concentration of 42% and further incubated for an hour and cold again. Then, the mixture was centrifuged at 16,000× *g* for 45 min at 4 °C. The supernatant and the pellet were separated, and the pH of the supernatant was reduced to 5.7 by the addition of 0.6 M cold sodium acetate solution, and it was incubated for an hour at 4 °C. The mixture is centrifugate at 16,000× *g* which yielded the second pellet. The pellets obtained were combined and resuspended in ammonium bicarbonate solution for tryptic peptide generation, and the supernatants with abundant albumin were discarded. The remaining serum proteins were separated into peptides by trypsin incubation, the released peptides were separated using nano-liquid chromatography and reverse-phase chromatography methods, and peptide sequence analysis and quantitation were performed on the QTOF/MS [21].

### 2.6. Statistical Methods

In this study, the clinical, laboratory, and cardiological examination results were compared using unpaired t-test for two-group comparisons (stage A versus stage B2, C and D, and pre- and post-treatment). Results are given as mean ± standard error, and *p* < 0.05 was considered statistically significant (SigmaStat 12.0, GmBH, Witzenhausen, Germany). The UniProt database was used for protein identification of *Canis lupus familiaris* (3 September 2021). Progenesis QIP software (V 4.1) was performed for protein identification and statistical analysis. Samples of each different stage of diseased dogs (B2, C and D) were compared to healthy dogs (stage A), and the proteins that showed a significant difference (*p* < 0.05) and change in concentrations higher or lower than 1.4-fold between groups were considered as up- or down-regulated. The fold change preferred for this study follows the example of our [21] and other previous studies of dogs with DCM [20]. Serum samples obtained before and after treatment of dogs at stage C of CHF were used to evaluate the effect of treatment on serum protein composition.

Bioinformatic analysis was performed for the mapping of protein–protein interaction, and the roles of proteins in molecular, cellular, and biological process (www.string-db.org, accessed on 3 September 2021 and http://pantherdb.org, respectively, accessed on 3 September 2021) [21]. Network representation of GO term of the proteins that were identified with statistically significant changes (at least *p* < 0.05) in dogs with CHF was created with the Euler diagram by open-source GOnet web application (https://tools.dice-database.org/GOnet/, accessed on 3 September 2021), as suggested by Pomaznoy et al. [25].

## 3. Results

Selected clinical, hematological, serum biochemical and echocardiographic characteristics observed in the dogs studied are presented in the Supplementary Materials Tables S1 and S2.

In this study, a total of 157 proteins were identified and quantified, with 11 being up-regulated and 21 down-regulated in dogs when B2, C, and D stages were compared to healthy control (stage A). Details of the proteomes such as accession numbers and fold changes are presented in Table 1.

In stage B2 dogs, angiotensinogen (AGT) was up-regulated, but immunoglobulin iota chain-like, lipopolysaccharide-binding protein (LBP), and carboxypeptidase (CPN) were down-regulated compared to stage A.

In stage C dogs, CPN, argininosuccinate lyase, complement C3 (C3), and inter-alpha-trypsin inhibitor heavy chain (ITIH) H2 and H4 were up-regulated, but complement proteins (C4, C7, C8, and factor D and I), hemopexin, apolipoprotein C-II, pigment epithelium-derived factor and actin-cytoplasmic-1 (ACT-1) were down-regulated compared to stage A.

In stage D, LBP, AGT and nuclear mitotic apparatus protein-1 were up-regulated, but serum albumin precursor, immunoglobulin lambda-1 light chain (IGLC1), tetranectin, paraoxonase-1 (PON-1), fetuin-B, adiponectin, platelet basic protein precursor, and ACT-1 were down-regulated compared to stage A.

A standard treatment protocol produced a decrease in serum CPN, complement C3 and AGT, and an increase in ACT-1 in dogs with stage C CHF.

**Figure 1:**
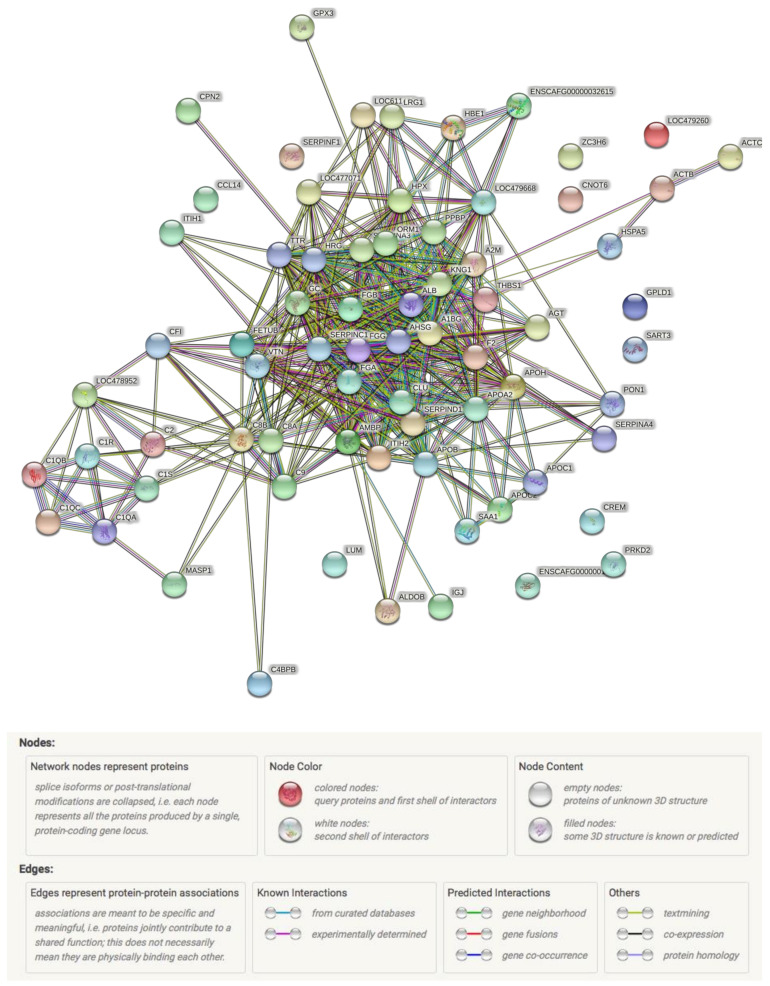
String interaction analysis of up- and down-regulated serum proteins in dogs with CHF compared to healthy controls. Legend: This figure was created using the open-source web application www.string-db.org (accessed on 3 September 2021).

**Figure 2:**
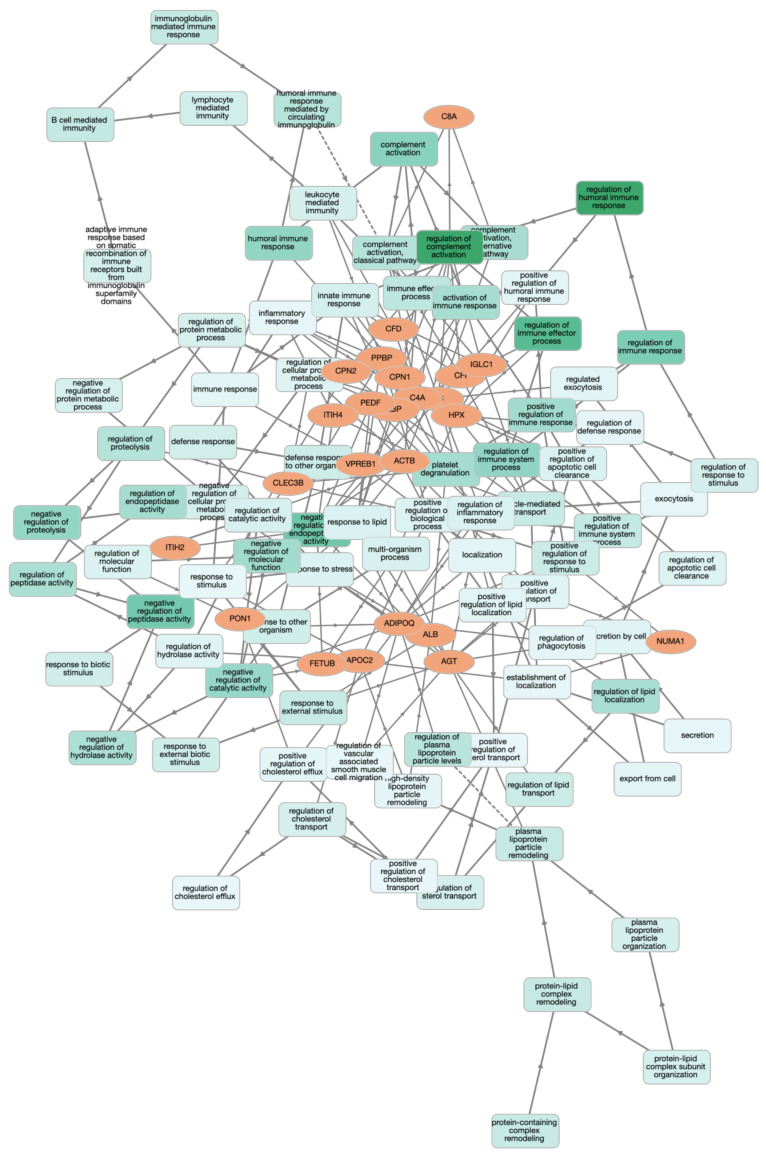
Network representation of Go term of serum proteins which showed the differences at statistically significant level in dogs with CHF compared to healthy controls. Legend: Short name of up- and down-regulated proteins and their biological functions are shown in the orange and green boxes, respectively. When the proteins have the same biological functions, light green is changed to dark green. This figure was created with the Euler diagram using the open-source GOnet web application (https://tools.dice-database.org/GOnet/, accessed on 3 September 2021).

String analysis showed the protein–protein interaction among identified proteins in this study (Figure 1); complement proteins and fibrinogen subtypes showed intense interactions among themselves. There were also interactions between fibrinogen, albumin, serpins, ITIH, fetuin, clusterin, apolipoproteins, and alpha-glycoprotein. The GO term results indicated that the identified proteins played roles especially in immune regulation, metabolic processes, and inflammatory responses (Figure 2). Panther Go-Slim results revealed molecular (catalytic and binding activity) and biological functions (immune system and metabolic processes) and pathway analysis (inflammation, cadherin signaling, nicotinic acetylcholine receptor signaling and Wnt signaling, etc.) for the proteins, showing the statistically significant changes in dogs at stage B2, C and D, compared to those at stage A (Supplementary File S3).

## 4. Discussion

In this study, a total of 32 different serum proteins showed changes in concentration at different stages of CHF compared to healthy dogs. These proteins are involved in a plethora of cellular, biological, and molecular processes, such as immune-inflammatory reaction, acute phase response, coagulation, and oxidative metabolism, which are suggestive of a role of these mechanisms in the pathogenesis of CHF. In addition, proteins that showed differences between the groups may be considered to be potential serum biomarkers for the severity of CHF, and proteins that changed significantly after a conventional treatment may be used as biomarkers for treatment monitoring of this condition.

In this study, some proteins (hemopexin—HPX, tetranectin, apolipoprotein, adiponectin, inter-alpha-trypsin inhibitor heavy chain—ITIH, complement, angiotensinogen—ANG, and paraoxanase 1—PON-1) identified in serum of the dogs with CHF due to MMVD had already been identified in previous studies performed in dogs with MMVD [10,11,12,13] and idiopathic DCM [20,21]. In parallel to the changes in serum proteins identified in those studies, serum levels of ITIH, complement, and ANG were found to be increased, whereas serum levels of HPX, tetranectin, apolipoprotein, adiponectin and PON-1 were found to be decreased in the present study.

Herein, we discussed the identified proteins according to the severity of CHF.

In stage B2, the up-regulation of angiotensinogen (AGT) found may be associated with the activation of the sympathetic system [20]. It was also up-regulated with a similar fold change in stage D, indicating that AGT is involved in different stages of severity, and therefore may not be a suitable biomarker to evaluate disease severity in CHF.

Immunoglobulin iota chain-like (VPREB1), lipopolysaccharide-binding protein (LBP), and carboxypeptidase (CPN2) were down-regulated in stage B2 dogs. VPREB1 regulates immunoglobulin gene rearrangements in the early steps of B-cell differentiation [26] and activates the B-cell signaling pathway. Although this protein has never been reported in dogs, it may be speculated that low concentrations of this immunoglobulin can lead to an impairment of immune response in the progression of CHF in this species. CPN protects the body from inflammatory peptides (kinins) and anaphylatoxins (i.e., C3), as well as the heart from pressure overload [27,28]. The decrease of CPN2 found in the B2 dogs could be due to its consumption as a protective reaction against hemodynamic stress and complement activation.

LBP concentrations decreased in stage B2 dogs compared to healthy controls, but was elevated in stage D. LBP is an acute-phase protein [29,30], and therefore it could be hypothesized that the increase of LBP at stage D may indicate a more severe inflammatory status, reflecting the poorer clinical condition appearing at this stage.

In stage C dogs, C3, a member of the complement system, was up-regulated, whereas other complement proteins (i.e., C4a, C7, and C8) were down-regulated, indicating a pathophysiological involvement of the complement system. The increases in C3 in human patients with CHF is related to chronic immune-inflammatory activation, leading to adverse left ventricular remodeling and aggravation of heart failure [31,32], and poorer outcome [31,33]. The role of complement proteins, as potent therapeutic and prophylactic targets to slow the progression of CHF, should be explored in the future.

In the present study, two antioxidant proteins, HPX and PEDF, were down-regulated at stage C. HPX is mainly synthesized in the liver and minimizes tissue injury and facilitates tissue repair in dogs and humans with various inflammatory diseases [34]. It shows antioxidative effects by protecting cells from the deleterious effects of ROS, free hemoglobin, and heme [35], as well as supporting vascular nitric oxide hemostasis [34]. Decreased HPX levels lead to systolic dysfunction due to free heme accumulation in the heart [36]. Thus, the observed decrease in serum HPX in stage C dogs in this study may be related to a pathophysiological reaction in response to tissue injury, ongoing inflammation and/or oxidative stress reported in our [11] and other previous studies [37,38,39,40,41]. Low serum HPX activity in dogs with CHF could be considered to be a biomarker of poor prognosis, as reported in humans with CHF [42].

In stage D, LBP was the protein that showed the highest up-regulation. Similar to serum CRP, LBP production increases due to pro-inflammatory cytokines in inflammatory states such as sepsis and multiple organ failure [43]. Thus, the observed increases in serum LBP may be an indicator for the activation of acute-phase reaction, possible due to bacterial translocation from the intestinal lumen into the bloodstream in dogs with stage D CHF. High serum LBP may be used as an indicator of CHF severity, as reported in humans with cardiovascular diseases [29,44].

IGLC was down-regulated in stage D. An alteration of this protein has been associated with heart failure in humans [45]. In the present study, decreased IGLC may have resulted from decreased B-cell activity, and could contribute to the impairment of immune-inflammatory responses that occurs with the progression of CHF.

PON-1 was one of the down-regulated proteins in serum samples of stage D dogs. This result agrees with the measurement of PON-1 in these dogs by spectrophotometric assays [11]. PON-1 is synthesized primarily from liver, and shows an antioxidative effect by protecting lipoproteins from the detrimental effects of ROS. In a previous study, an increase in oxidative stress and production of ROS in different stages of heart failure (according to NYHA classification) were associated with decreasing PON-1 activity, that is, as disease severity increased, serum PON-1 activity decreased gradually in dogs with CHF [39]. Thus, observed low levels of PON-1 in stage D may indicate decreasing antioxidant capacity, as well as the severity of CHF in dogs. In addition, serum PON-1 is a negative acute-phase protein in dogs and humans with inflammatory diseases [35,46,47]. The presence of tissue injury, inflammation, and/or oxidative stress during disease progression could lead to decreased serum PON-1 in dogs [39]. Low serum PON-1 activity may be a risk factor for long-term adverse cardiac events in dogs, as reported in humans with CHF [48].

Adiponectin is the most abundant adipokine produced by white adipose tissue, and acts as a negative acute-phase protein in dogs [49]. Adipokines affect cardiovascular functions and several physiological processes, such as energy metabolism, immune function, and inflammation [10,12,50]. The observed decrease in serum adiponectin in stage D in this study parallels the results of dogs with MMVD [12], but was in contrast to other studies in dogs with DCM [51] and MMVD [52]. Since high adiponectin levels are associated with low cardiovascular risk and slowing the progression of cardiovascular diseases in humans [10,53,54], low serum adiponectin may reflect the disease severity of CHF and may be a risk factor for poor outcome in end-stage CHF in dogs.

Tetranectin (TN), a regulator of the fibrinolysis and proteolytic systems, was down-regulated in stage D dogs, as reported in previous studies in dogs with CHF [12] and humans with cardiovascular disease [55,56]. Our findings may support a recent study reporting that low TN levels were associated with CHF more closely than B-type natriuretic peptide in humans [57], suggesting that TN may be a biomarker candidate for heart failure in dogs.

In the present study, following treatment, two proteins (CPN and C3) were up-regulated, and seven proteins (i.e., albumin precursor, AGT, ACT-1, and fetuin-B) were down-regulated, showing the treatment-related changes in serum proteome patterns of dogs with CHF in parallel to improvement of their clinical conditions. Conventional treatment for heart failure could attenuate the changes in up- (CPN1, and C3) and down-regulated proteins (serum albumin precursor, albumin isoform X1, IGLC, and fetuin-B) in stage C CHF. Our results indicate that these proteins, such as CPN, C3, AGT and ACT-1, which showed significant changes after the standard therapy for CHF, can be used for disease monitoring and may relate to beneficial effects of the medication. Although the dogs received medication (ACE-i) for the control of hypertension, serum AGT concentrations were still high after treatment. Therefore, AGT seems not to be a valid biomarker of disease progression or treatment monitoring.

Based on the bioinformatic analysis, protein–protein interactions between complement proteins, fibrinogen subtypes, and others (albumin precursor, serpins, ITIH, fetuin, clusterin, apolipoproteins, and alpha-glycoproteins) showed that the pathophysiology of CHF seems to be more sophisticated than we had previously known. Their molecular and biological functions as well as roles in signaling pathways, such as inflammation [11], cadherin signaling [58], nicotinic acetylcholine receptor signaling [59], and Wnt signaling [60], make them possible biomarkers and therapeutic targets for the diagnosis and treatment of dogs with CHF.

This study has several limitations. Since there were small sample sizes in each group, this study can be considered to be a pilot study. The differences in body weight, sex, and age between groups may affect the protein signature in dogs, as reported in healthy humans [61]. Despite these limitations, our findings open up a new area of research. Future studies are needed to replicate and expand on our findings in a larger population with age- and sex-matched case and control groups.

## 5. Conclusions

This study revealed that dogs with different stages of CHF show different serum protein composition. These proteins are associated with several cellular, biological, and metabolic processes such as immune-inflammatory responses, hemostasis, oxidative stress, and energy metabolism, which might be detrimental in the progression of canine CHF. These proteins indicate different pathophysiological changes occurring at the different stages of CHF, and could be potential biomarkers for diagnosis and treatment monitoring of this condition.

## Figures and Tables

**Table 1:** Protein expressions with significantly differential abundances between the groups of dogs with different stages of CHF.

Protein Expressions between Stage A and B2			
Accession Number *	Protein	Fold Change	Description
**XP_005618890.1**	Angiotensinogen	1.62	Up-regulated
**XP_022266499.1**	Immunoglobulin iota chain-like	24.48	Down-regulated
**XP_542993.3**	Lipopolysaccharide-binding protein	1.72	Down-regulated
**XP_005639693.1**	Carboxypeptidase N subunit 2	1.58	Down-regulated
**Protein Expressions between Stage A and C**			
Accession Number *	**Protein**	**Fold Change**	**Description**
**XP_534989.4**	Carboxypeptidase N catalytic chain isoform X1	3.09	Up-regulated
**XP_005633270.2**	Complement C3	2.76	Up-regulated
**XP_013969620.1**	Argininosuccinate lyase isoform X2	2.75	Up-regulated
**XP_005642305.2**	Inter-alpha-trypsin inhibitor heavy chain H4 isoform X1	2.58	Up-regulated
**XP_848765.4**	Inter-alpha-trypsin inhibitor heavy chain H4 isoform X3	2.58	Up-regulated
**XP_535195.2**	Inter-alpha-trypsin inhibitor heavy chain H2	1.76	Up-regulated
**XP_013977971.1**	Nuclear mitotic apparatus protein 1 isoform X2	1.64	Up-regulated
**XP_022273600.1**	Complement component C7 isoform X2	2.77	Down-regulated
**XP_853676.1**	Hemopexin	2.46	Down-regulated
**XP_013977853.1**	Complement factor D isoform X1	1.90	Down-regulated
**NP_001003368.1**	Apolipoprotein C-II precursor	1.75	Down-regulated
**NP_001071056.2**	Pigment epithelium-derived factor	1.68	Down-regulated
**XP_022281488.1**	Complement C4-A	1.67	Down-regulated
**XP_003639070.1**	Complement component C8 alpha chain isoform X1	1.67	Down-regulated
**XP_005639325.1**	Complement factor I isoform X3	1.57	Down-regulated
**NP_001182774.2**	Actin_ cytoplasmic 1	1.47	Down-regulated
**Protein Expressions between Stage A and D**			
Accession Number *	**Protein**	**Fold Change**	**Description**
**XP_542993.3**	Lipopolysaccharide-binding protein	3.53	Up-regulated
**XP_005618890.1**	Angiotensinogen	1.54	Up-regulated
**XP_013977971.1**	Nuclear mitotic apparatus protein 1 isoform X2	1.52	Up-regulated
**NP_001003026.1**	Serum albumin precursor	4.97	Down-regulated
**XP_005628024.1**	Serum albumin isoform X1	4.97	Down-regulated
**XP_022266304.1**	Immunoglobulin lambda-1 light chain isoform X38	4.49	Down-regulated
**XP_850219.1**	Serum paraoxonase/arylesterase 1	2.91	Down-regulated
**XP_013977261.1**	Tetranectin	2.91	Down-regulated
**XP_535835.2**	Fetuin-B	2.91	Down-regulated
**NP_001165243.2**	Platelet basic protein precursor	2.38	Down-regulated
**NP_001182774.2**	Actin_ cytoplasmic 1	1.96	Down-regulated
**XP_022269775.1**	Adiponectin isoform X2	1.74	Down-regulated
**Protein Expressions of Post-Treatment Patients in Stage C Compared to Stage A**			
Accession Number *	**Protein **	**Fold Change**	**Description**
**XP_534989.4**	Carboxypeptidase N catalytic chain isoform X1	2.12	Up-regulated
**XP_005633270.2**	Complement C3	1.77	Up-regulated
**NP_001003026.1**	Serum albumin precursor	3.53	Down-regulated
**XP_005628024.1**	Serum albumin isoform X1	3.53	Down-regulated
**XP_022266304.1**	Immunoglobulin lambda-1 light chain isoform X38	2.69	Down-regulated
**XP_005618890.1**	Angiotensinogen	2.58	Down-regulated
**NP_001182774.2**	Actin_ cytoplasmic 1	2.20	Down-regulated
**XP_535835.2**	Fetuin-B	2.19	Down-regulated
**XP_536110.2**	complement factor H	1.87	Down-regulated

* Accession number from NCBI protein database for *Canis lupus familiaris*.

## Data Availability

Data is contained within the article or Supplementary Material. The data presented in this study are available in [https://www.mdpi.com/article/10.3390/ani12040490/s1].

## Supplementary Materials

The following supporting information can be downloaded at: https://www.mdpi.com/article/10.3390/ani12040490/s1, Supplementary File S1: Some clinical, hematological, serum biochemical and echocardiographic parameters (mean ± SEM) in dogs with different stages of CHF (ACVIM consensus, Keene et al., 2019), Supplementary File S2: Pre- and post-treatment results of some clinical, hematological, serum biochemical and echocardiographic parameters (mean ± SEM) in dogs with CHF in stage C (ACVIM Consensus Guidelines, Keene et al., 2019), Supplementary File S3: Molecular and biological functions, cellular component, protein class and pathway analysis of the proteins showing statistically significant fold changes in dogs with CHF compared to those of healthy controls.
