# Supplementary material for: Serum Proteomic Changes in Dogs with Different Stages of Chronic Heart Failure
Source: Animals (Basel). 2022 Feb 16;12(4):490. doi: 10.3390/ani12040490 (PMC8868296; doi:10.3390/ani12040490)
Supplement: Supplementary file 1 [file animals-12-00490-s001.zip › animals-1506268-supplementary.pdf]

Supplementary File S1. Some clinical, hematological, serum biochemical and echocardiographic parameters (mean ± SEM) in dogs with different stages of CHF (ACVIM Consensus Guidelines, Keene et al., 2019).

**Parameter.**	**Stage A (n = 8)**	**Stage B2 (n = 8)**	**Stage C (n = 8)**	**Stage D (n = 8)**
**Clinical parameters**				
**PR** /min	119 ± 31^{a}	128 ± 23^{ab}	156 ± 18^{abc}	$191 \pm 72^{c***,\#**}$
**BR** /min	18 ± 10^{a}	$19 \pm 6^{b**}$	$68 \pm 14^{b***}$	$63 \pm 19^{b**}$
**Hemobiochemical parameters**				
**Hct** %	43.8 ± 3.2ab	48.8 ± 5.3a	43.6 ± 9.2ab	37.6 ± 7b***
**CRP** μg/mL	2,1 ± 3,2a	4,3 ± 4,2a	10,1 ± 4,5ab	37,8 ±13,9b**
**cTnI** ng/mL	0.03 ± 0.04a	0.03 ± 0.04a	1.80 ± 1.39b*	7.09 ± 12.2b*
**BUN** mg/dL	14.1 ± 4.3a	15.1 ± 6.1a	22.3 ± 15.5a	34.5 ± 24.2a
**Cr** mg/dL	0.8 ± 0.2a	0.8 ± 0.07a	0.91 ± 0.3a	1 ± 0.25a
**Echocardiographic parameters**				
**LA/Ao**	1.1 ± 0.1a	1.6 ± 0.1b	2.2 ± 0.5b***	2.3 ± 0.5bc***
**LVIDDn**	1.5 ± 0.2a	1.8 ± 0.2ab	2.3 ± 0.3bc	3.0 ± 0.4d***#**
**FS** %	32.2 ± 6.0a	35.0 ± 5.2a	29.5 ± 9.0a	26.7 ± 14.6a
**MV E/A ratio**	1.5 ± 0.4a	2.0 ± 0.4b*	2.2 ± 0.6b*	3.8 ± 1.4b**
**Medication**				
**Pimobendan**	0/8	8/8	8/8	8/8
**Enalapril/Ramipril**	0/8	0/8	8/8	8/8
**Furosemide**	0/8	0/8	8/8	4/8
**Hydrochlorothiazide**	0/8	0/8	0/8	8/8
**Spironolactone**	0/8	0/8	0/8	5/8
**Torasemide**	0/8	0/8	0/8	4/8
**Antiarrhythmic +**	0/8	0/8	0/8	4/8

* P < 0.05   ** P < 0.01   *** P < 0.001

# Compared to stage B2

PR- Pulse Rate, B**R-** Breath Rate, **Hct**- Hematocrit, **CRP**- Serum C-reactive protein, **BUN**- Blood urea nitrogen, **Cr**- Creatinine, **LA/Ao-** aorta to left atrium ratio, **LVIDDn**, normalized left

ventricular internal dimention in diastole, **FS-** fractional shortening, **MV E/A** – Mitral valve E and A velocity

+ Digoxin (0.005 – 0.008 mg/kg, q12 hr, PO) and/or diltiazem (0.5-1 mg/kg, q8-12hr, PO).

Supplementary File S2. Pre- and post-treatment results of some clinical, hematological, serum biochemical and echocardiographic parameters (mean ± SEM) in dogs with CHF in stage C (ACVIM Consensus Guidelines, Keene et al., 2019).

**Parameter.**	**Pre-treatment (n = 8)**	**Post-treatment$^{\Psi}$ (n = 8)**
	**Clinical parameters**	
**PR** /min	156 ± 18^{abc}	144 ± 16**
**BR** /min	68 ± 14^{b***}	28 ± 14***
	**Hemobiochemical parameters**	
**Hct** %	43.6 ± 9.2ab	42.6 ± 6.5
**CRP** μg/mL	10,1 ± 4,5ab	3,3 ± 2,2***
**cTnI** ng/mL	1.80 ± 1.39b*	1.10 ± 0.91
**BUN** mg/dL	22.3 ± 15.5a	30.7 ± 26.0
**Cr** mg/dL	0.91 ± 0.3a	0.84 ± 0.33
	**Echocardiographic parameters**	
**LA/Ao**	2.2 ± 0.5b***	1.9 ± 0.4
**LVIDDn**	2.3 ± 0.3bc	2.0 ± 0.2
**FS** %	29.5 ± 9.0a	33.4 ± 8.2
**MV E/A ratio**	2.2 ± 0.6b*	1.9 ± 0.6
	**Medication**	
**Pimobendan**	8/8	8/8
**Enalapril/Ramipril**	8/8	8/8
**Furosemide**	8/8	8/8
**Hydrochlorothiazide**	0/8	3/8$^{\epsilon}$
**Spironolactone**	0/8	3/8$^{\epsilon}$
**Torasemide**	0/8	0/8
**Antiarrhythmic +**	0/8	2/8$^{\epsilon}$

* P < 0.05   ** P < 0.01   *** P < 0.001

$^{\Psi}$ compared to stage C before treatment $^{\epsilon}$ added according to requirement of the dogs based on the cardiac observations (radiography, ECG, and echocardiography) during re-examinations, two weeks later from initial treatments.

PR- Pulse Rate, B**R**- Breath Rate, **Hct**- Hematocrit, **CRP**- Serum C-reactive protein, **BUN**- Blood urea nitrogen, **Cr**- Creatinine, **LA/Ao**- aorta to left atrium ratio, **LVIDDn**, normalized left ventricular internal dimention in diastole, **FS-** fractional shortening, **MV E/A** – Mitral valve E and A velocity

+ Digoxin (0.005 – 0.008 mg/kg, q12 hr, PO) and/or diltiazem (0.5-1 mg/kg, q8-12hr, PO).

Supplementary File S3. Molecular and biological functions, cellular component, protein class and pathway analysis of the proteins showing statistically significant fold-changes in dogs with CHF compared to those of healthy controls.

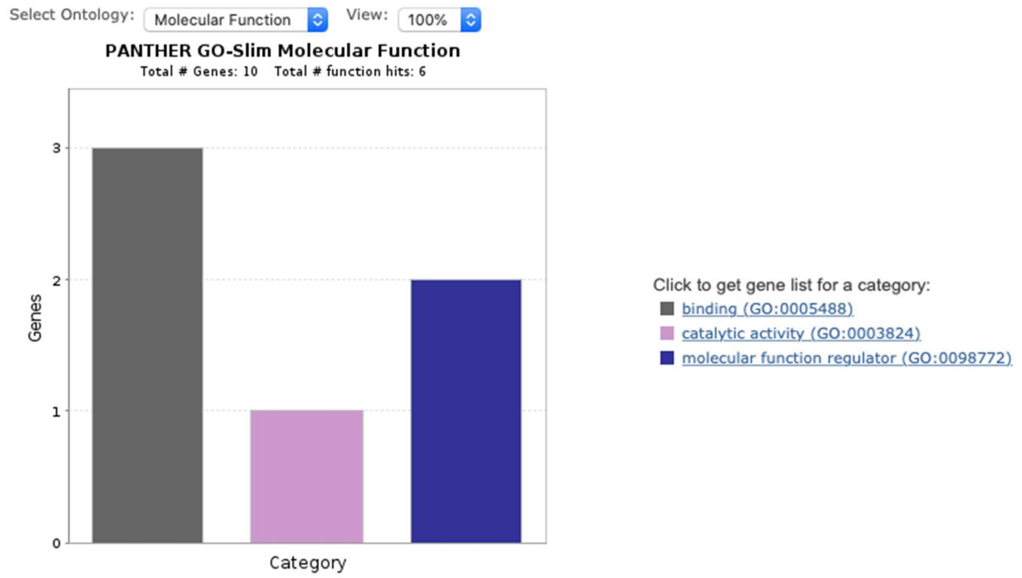

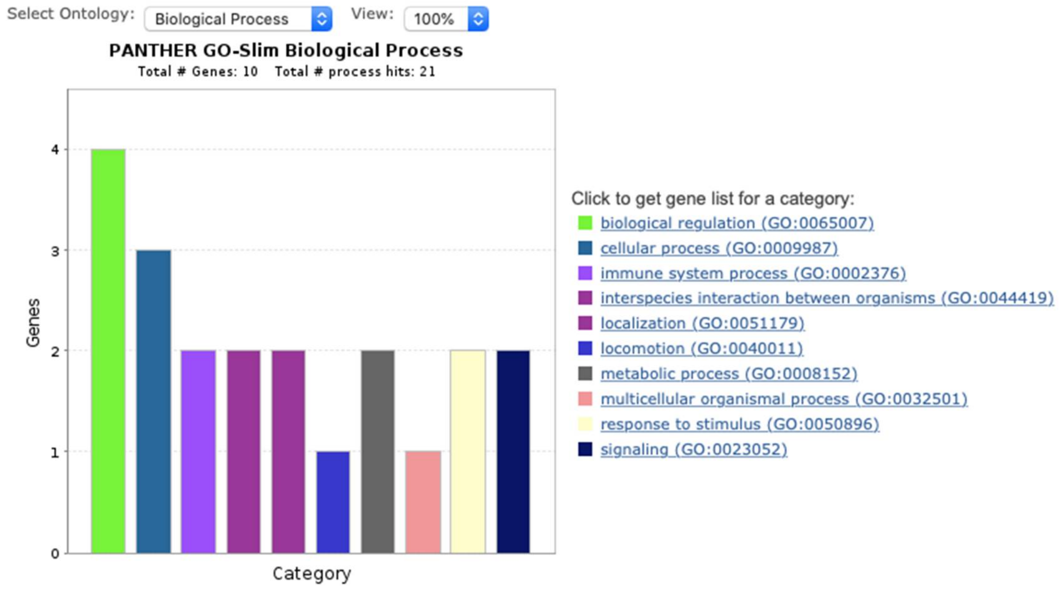

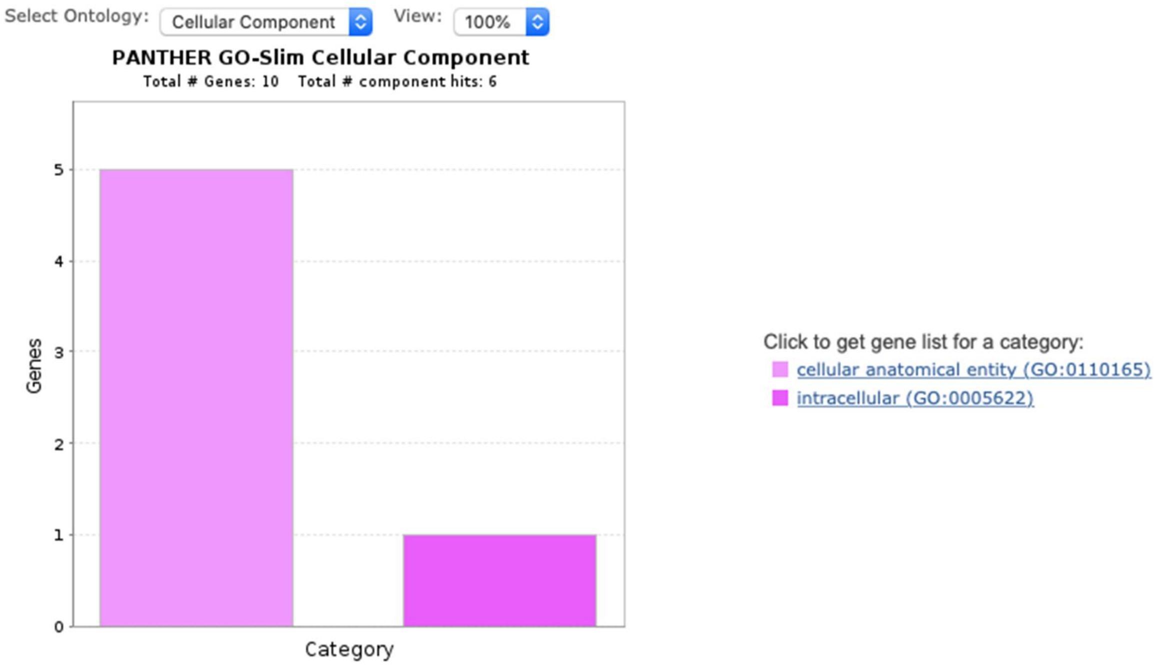
Select Ontology: Cellular Component View: 100%
PANTHER GO-Slim Cellular Component
Total # Genes: 10 Total # component hits: 6
Genes
0
1
2
3
4
5
Category
Click to get gene list for a category:
cellular anatomical entity (GO:0110165)
intracellular (GO:0005622)

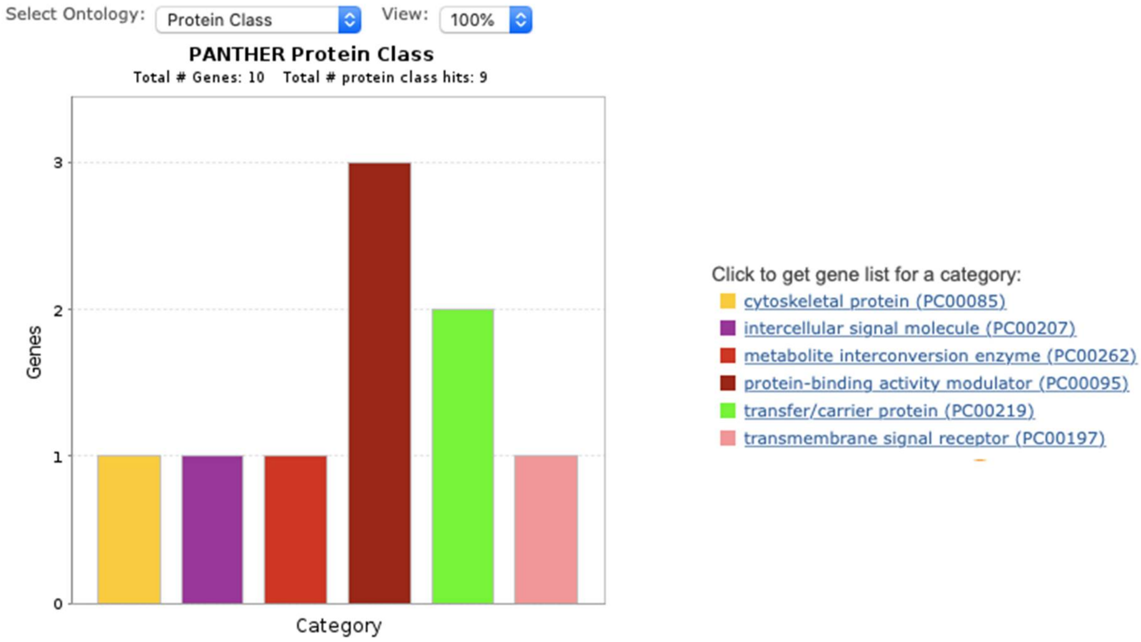
Select Ontology: Protein Class View: 100%
PANTHER Protein Class
Total # Genes: 10 Total # protein class hits: 9
Genes
0
1
2
3
Category
Click to get gene list for a category:
cytoskeletal protein (PC00085)
intercellular signal molecule (PC00207)
metabolite interconversion enzyme (PC00262)
protein-binding activity modulator (PC00095)
transfer/carrier protein (PC00219)
transmembrane signal receptor (PC00197)
